# Competing Endogenous RNA: The Key to Posttranscriptional Regulation

**DOI:** 10.1155/2014/896206

**Published:** 2014-02-02

**Authors:** Rituparno Sen, Suman Ghosal, Shaoli Das, Subrata Balti, Jayprokas Chakrabarti

**Affiliations:** ^1^Gyanxet, BF 286 Salt Lake, Kolkata, West Bengal 700064, India; ^2^Indian Association for the Cultivation of Science, Kolkata, West Bengal 700032, India

## Abstract

Competing endogenous RNA, ceRNA, vie with messenger RNAs (mRNAs) for microRNAs (miRNAs) with shared miRNAs responses elements (MREs) and act as modulator of miRNA by influencing the available level of miRNA. It has recently been discovered that, apart from protein-coding ceRNAs, pseudogenes, long noncoding RNAs (lncRNAs), and circular RNAs act as miRNA “sponges” by sharing common MRE, inhibiting normal miRNA targeting activity on mRNA. These MRE sharing elements form the posttranscriptional ceRNA network to regulate mRNA expression. ceRNAs are widely implicated in many biological processes. Recent studies have identified ceRNAs associated with a number of diseases including cancer. This brief review focuses on the molecular mechanism of ceRNA as part of the complex post-transcriptional regulatory circuit in cell and the impact of ceRNAs in development and disease.

## 1. Introduction

**Figure 1:**
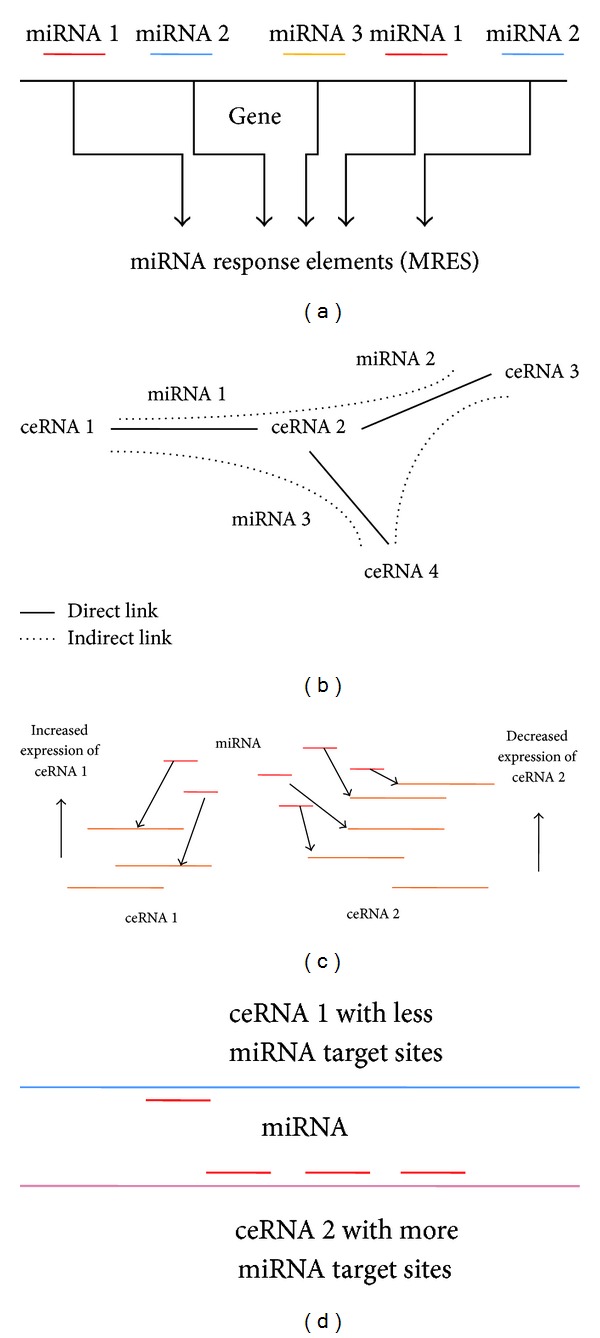
Determinants of ceRNA activity. (a) The direct and indirect linkage between ceRNAs in a network of four ceRNAs. ceRNA 1 and ceRNA 2 are directly linked by the common miRNA, miRNA 1, and ceRNA 2 and ceRNA 3 are linked by the common miRNA, miRNA 2. ceRNA 1 and ceRNA 3 are indirectly linked through ceRNA 2. (b) The effect of concentration of individual ceRNAs. ceRNA 2 has a higher level of expression than ceRNA 1 which drives the shared miRNAs more towards ceRNA 2 leaving ceRNA 1 free of repression. (c) The effect of number of MREs on ceRNAs for shared miRNAs. ceRNA 2 has more target sites for the shared miRNA (red) than ceRNA 1 so that the miRNA is more attracted to ceRNA 2. It results in increased ceRNA effect of ceRNA 2 on ceRNA 1.

microRNAs (miRNAs) are typically 22 nucleotides long and they negatively regulate, that is, repress, mRNAs or noncoding transcripts. A single mRNA can be regulated by a number of miRNAs, and a single miRNA can regulate multiple mRNAs [1, 2]. As each miRNA may repress a large number of transcripts, it is estimated that miRNAs regulate a large proportion of the transcriptome [2–4]. Every miRNA target contains MREs (miRNA response elements), to which miRNAs are attracted, resulting in down regulation of the target (Figure 1(a)). The miRNAs bind to partially complementary sequences in the 3′ untranslated region (UTR) of the target mRNA and result in its repression mediated by Argonaute (Ago) proteins [1, 4]. The concentration of the miRNA targets is an important factor that determines the availability of free miRNA within cells as higher concentration of miRNA-targets is indicative of lower availability of free miRNAs [5]. The activity of the miRNAs can be affected by the presence of competitive endogenous RNAs (ceRNAs), vying for the miRNAs with shared MREs [6, 7]. These ceRNAs act as modulators for a single miRNA or multiple miRNAs. The modulation is done through influencing the available levels of miRNAs in a cell. ceRNAs can act as a quality control factor regulating miRNA expression levels needed for adequate repression of the miRNA-target genes [7].

In recent studies, it has been discovered that pseudogenes, long noncoding RNAs (lncRNAs), and circular RNAs (circRNAs) can act as miRNA “sponges” by sharing common MREs, inhibiting normal miRNA activity [8–10]. These sponges “absorb” miRNAs, lowering the levels of available miRNAs for the target mRNA, resulting in increased translations. The first endogenous “sponge” RNA was discovered in plants in a situation where a stress related miRNA-mediated response was reduced [10].

The ceRNAs are implicated in many biological processes and the disruption of the equilibrium of ceRNAs and miRNAs can be critical for ceRNA activity and promotion of diseases like cancer. ceRNAs have been found to be important regulator in many types of cancer [11–13]. ceRNAs have also been found to be involved in developmental stages (e.g., linc-MD1 [9]). This review focuses on the molecular mechanism of ceRNAs as part of a complex posttranscriptional regulatory circuit in cells and the impact of ceRNAs in development and disease.

## 2. Molecular Mechanism of ceRNAs

The ceRNAs compete for miRNA binding sites and regulate each other in ceRNA networks (ceRNETs) [11–13]. These ceRNETs regulate miRNAs and the whole post-transcriptional gene expression regulatory network mediated by miRNAs [20]. Generally there are two types of connections between the component ceRNAs in a ceRNET: a direct linkage between two ceRNAs exists when they share MRE(s) for one or more common miRNA and an indirect linkage exists when they do not share common miRNAs but are linked with a common ceRNA (Figure 1(b)). The ceRNETs are very complex and densely interlinked where not only the common direct linkage but also the indirect linkage between the component ceRNAs imparts profound regulatory effect on the other. In a network of four ceRNAs, where there exists a direct connection between ceRNA 1 and ceRNA 2, regulating the expression level of ceRNA 1 has a direct effect on the expression level of ceRNA 2. Whereas if there is an indirect connection between ceRNA 1 and ceRNA 2 via ceRNA 3, severing the direct connection results in a minimal effect on the expression of ceRNA 2 when the expression levels of ceRNA 1 are modulated [20].

### 2.1. The Concentrations of the Components in a ceRNA Network Determine the ceRNA Activity

In a mathematical model aimed to simulate the behaviour of the components of a ceRNET in a system of M number of miRNAs interacting with N number of targets [21], authors observed a threshold like behaviour in the system as commonly seen in a titration mechanism. In this general network, it was seen that, if at least the expression of one ceRNA rises above the threshold, the other common mRNA targets are also expressed above the threshold. The available or free miRNA levels go down along with it as almost all of them are bound in complex. The authors showed that theoretically, near the threshold, such systems show hypersensitivity to the change in the levels of the participating molecules (i.e., transcription rates of ceRNAs and miRNAs). In their model they have studied the effects of instantaneous increase or decrease in expression of a particular ceRNA on another ceRNA by measuring how fast the latter reaches equilibrium after the perturbation (measured by its response time, i.e., time needed to reach halfway between its initial and final steady state). In their model they have shown the effects of increasing the transcription rate of the shared miRNA and, secondly, the number of ceRNAs [21]. Similar observation was made by Ala et al. [20] who proposed that a near equimolar equilibrium of all the components in a ceRNA network is required for optimal ceRNA activity. They validated their hypothesis by showing the dose-dependent cross regulation of PTEN and its ceRNA VAPA (VAMP associated protein A). The authors selected five cell lines where VAPA is expressed in higher levels than PTEN. It was showed that upon siRNA mediated knockdown of VAPA, the cell lines where the ratio of the basal expression levels of VAPA and PTEN was lower (i.e., concentration of VAPA and PTEN was nearly equimolar) exhibited more profound ceRNA effect on PTEN. It was shown that the relative basal levels of the ceRNAs are predictive of how they will cross regulate each other. The component with higher expression has a more prominent ceRNA effect on the component with lower expression as seen by the experimentation with VAPA and PTEN (Figure 1(c)).

The concentration of miRNAs is an important factor for ceRNA activity. If there are a less number of miRNAs than their targets, the ceRNA activity is reduced as the targets will remain largely unrepressed. Also, if there are more miRNAs as compared to their targets, there would have been no cross regulation due to almost a universal repression of the targets. In the study by Ala et al. [20], the authors demonstrated the effects of perturbation of the components in a network of three ceRNAs and one miRNA. They checked the effects on the ceRNA network when the levels of expression of the miRNA were modulated. A lower expression of the miRNA coupled with an increased transcription rate of one ceRNA has a minimal effect on the other two ceRNAs while a higher expression of the miRNA, with the same conditions, has a significant effect on the other ceRNAs. The authors have showed that, when the expression of one ceRNA is increased, the levels of the other two ceRNAs have risen along with a fall in available miRNA level indicating that regulating the expression level of one ceRNA induces a profound effect on the whole network.

### 2.2. Other Determinants of ceRNA Activity

Other than the relative levels of the components of a ceRNA network, there are other determinants of ceRNA activity. One very important determinant is the number of shared miRNAs between two ceRNAs. Ala et al. showed that, in a network of 10 ceRNAs and 10 miRNAs, silencing of a ceRNA had greater impact on the components that shared more common miRNAs while it had negligible impact on the ceRNAs that were targeted by nonshared miRNAs [20].

### 2.3. ceRNA Database

Sarver and Subramanian [22] have developed a putative human ceRNA database ceRDB, which stores information about genes that can act as ceRNAs competing for same miRNA(s). They procured putative conserved miRNA-mRNA target interactions from TargetScan (http://www.targetscan.org/). There are practically numerous conserved miRNA binding sites within the mRNA transcriptome as predicted by TargetScan and only a small part of it is validated. In ceRDB, the competing mRNAs are sorted by an interaction score based on the number of shared MREs between the ceRNAs. The higher the score is, the higher the possibility of the target mRNA being affected by the presence of those ceRNAs is. The database ceRDB helps us get a picture of the potential ceRNA networks in human. But one shortcoming of ceRDB is that it considers only mRNAs with shared MREs for the ceRNA network, while it is evident that, other than just mRNAs, many noncoding transcripts act as ceRNAs. A recently developed database (available from http://gyanxet-beta.com/) provides information about lncRNAs that potentially act as ceRNAs. This database includes human miRNA targets on GENCODE 19 annotated lncRNAs [23] and lists the potential ceRNAs for a given mRNA or lncRNA sorted by the number of shared miRNAs between them. Unlike ceRDB which contains purely putative miRNA-mRNA interactions, this new database includes miRNA targets on mRNAs and lncRNAs mapped into Ago interacting regions. Importantly, driven by the observation of Ala et al. [20] that near equimolarities of the ceRNA components imparts the most profound effect on each other, this database gives users a provision so that they can judge the potential ceRNA effect on a pair of ceRNAs by checking the relative expression levels of the pair along with co-expressing shared miRNAs over different tissues.

## 3. ceRNA in Development

Given the complex regulatory network controlling the gene expression in development and the well known involvement of miRNAs, it is clear that ceRNA circuitry is involved. There is good deal of evidence of network of ceRNAs in embryonic stem cells and in muscle development. The existing evidence shows involvement of lncRNAs in the ceRNA activity which is an interesting phenomenon. lncRNAs are known players in development [24], but till now most lncRNAs are found to be associated with epigenetic modifications [25]. But given the fact that many lncRNAs contain miRNA binding sites in them [26], it is clear that a large number of them function as ceRNAs in developmental stages.

The study of Cesana and his group has suggested that linc-MD1, a muscle-specific long noncoding RNA, plays an important role in muscle development by a ceRNA function. linc-MD1 blocks miR-133 from binding to MAML1 and MEF2C, the transcription factors involved in myogenic differentiation, resulting in freeing the mRNAs from miR-133 mediated repression. This phenomenon activates muscle-specific gene expression leading to myogenic differentiation [9].

There are many reports of involvement of lncRNAs in pluripotency (recently reviewed by Ghosal et al. [27]). linc-RoR (regulator of reprogramming), a lincRNA found to be enriched in human induced pluripotent stem cell (iPSC) and embryonic stem cell (ESC), was recently identified as a key component in the ceRNA network in ESCs. Previously Rinn and colleagues showed that this lincRNA is regulated by Oct4 in human iPSCs and plays a role in iPSC colony formation [24]. Recently Wang et al. [28] found that linc-RoR shares common MRE with the core transcription factors (TFs) in ESC, namely, Oct4, Sox2, and Nanog, and reduces the miRNA-mediated repression of these TFs by sequestering miR-145. The authors indicated that the ceRNA activity results in a feedback regulatory loop of the linc-RoR with the core ESC TFs as they cross regulate each other to lift their level in a typical ceRNA mechanism. They suggested that this feedback regulatory loop is operating in ESC for maintenance of pluripotency.

## 4. ceRNA in Disease

It is well known that the disruption of the equilibrium of ceRNAs and miRNAs can be critical for ceRNA activity in diseases like cancer. ceRNAs have influential roles in diseases by regulating expressions of disease related genes. The most pronounced evidence of disease associated ceRNAs is the case of tumor suppressor gene PTEN. PTEN is involved in cancer and is a negative regulator of the oncogenic phosphoinositide 3-kinase/Akt signalling pathway. Studies reveal that PTEN expression is controlled by ceRNA circuitry in prostate cancer, glioblastoma, and melanoma, and disruption in the network leads to tumorigenesis in many cases. Karreth and his group have successfully validated a protein-coding transcript ZEB2 as a PTEN ceRNA in melanoma. They showed that this PTEN ceRNA ZEB2 is involved in regulation of PTEN expression in a miRNA-dependent manner [11]. In another study, Tay and his group have showed that protein-coding transcripts CNOT6L and VAPA are involved in regulation of PTEN expression by a ceRNA mechanism in prostate cancer [12]. By computational analysis, Sumazin and his group [13] identified nearly 7000 genes acting as miRNA sponges in glioblastoma. This network of ceRNAs was found to regulate key players in tumorigenesis or tumor suppression including PTEN. For confirmation of their hypothesis they knocked down 13 predicted PTEN ceRNAs, which resulted in down-regulation of PTEN expression, in a 3′UTR-dependent manner, and increased tumor cell growth [13]. The study of Jeyapalan and his group has demonstrated that the 3′UTR of CD44 regulates miRNA mediated repression activity in human breast cancer cell line MT-1 by functioning as ceRNA. CD44 has been found to be associated with many cancers, and its upregulation is associated with favourable outcomes. They found that exogenous overexpression of CD44 3′-UTR resulted in increased translation of CD44 and CDC42, a gene involved in controlling migration and cell-cycle progression. They identified that miR-216a, miR-330, and miR-608 can bind 3′UTR of both CD44 and CDC42 [14]. In the study by Lee and his group, the authors showed that over-expression of the 3′UTR of the extracellular matrix associated gene versican (VCAN) leads to the cellular level reduction of one of its targeting miRNAs, miRNA-199a-3p. Versican is involved in cell adhesion, proliferation, migration, and angiogenesis. Their computational analysis indicated that, among the putative targeting miRNAs of versican 3′UTR, miR-199a-3p and miR-144 targeted a cell cycle regulator Rb1 and miR-144 and miR-136 were found to target PTEN. Further, it was shown that over-expression of versican 3′UTR in breast cancer cell line 4T1 triggered the up-regulation of Rb1 and PTEN expression in vitro and in vivo. These results indicated that the versican 3′UTR modulates activities of miRNAs targeting Rb1 and PTEN by binding them which results in subsequent freeing of Rb1 and PTEN mRNAs for translation [15].

Poliseno and his group suggested that PTENP1, a non-protein-coding pseudogene of PTEN, has certain role in changing PTEN expression. It sequesters miR-19b and miR-20a and blocks them from binding to PTEN, controlling downstream PI3 K signalling and cell proliferation [8]. Another pseudogene shown to have ceRNA activity is that of the protooncogene KRAS, KRAS1P, which increases KRAS transcript abundance and accelerates cell growth [8].

### 4.1. The Activity of lncRNAs as miRNA Sponges

lncRNA HULC is one of the most upregulated of all genes in hepatocellular carcinoma. Wang and his group have demonstrated that the HULC lncRNA acts as ceRNA of the protein coding gene PRKACB and induces its increased translation by controlling the activity of miR-372. PRKACB induces activation of CREB (cAMP response element binding protein) which in turn is involved in up-regulation of HULC. This way HULC lncRNA modulates self-regulation in hepatocellular carcinoma by an autoregulatory loop involving CREB [16]. Fan et al. [17] investigated the role of the lncRNA PTCSC3 (papillary thyroid carcinoma susceptibility candidate 3) as ceRNA in thyroid cancer cell lines. PTCSC3 is a newly identified and highly thyroid specific lncRNA. The transfection of PTCSC3 resulted in significant growth inhibition in all tested thyroid cancer cell lines (BCPAP, FTC133, and 8505C). The authors bioinformatically predicted 20 miRNAs which have potential target sites in PTCSC3. Out of 20 miRNAs, miR-574-5p was selected to further confirm the inverse correlation of over-expression of PTCSC3 with growth of thyroid cancer cells in vitro. The authors noted significant down-regulation of endogenous miR-574-5p following PTCSC3 over-expression. They concluded that PTCSC3 may act as a tumor suppressor and a competing endogenous RNA for miR-574-5p [17]. A computational analysis by Arancio et al. for searching possible ceRNAs of the gene LMNA, involved in Hutchinson-Gilford progeria syndrome, identified 17 putative ceRNAs associated with 11 miRNAs. Gene ontology analysis of isolated ceRNAs showed enrichment in RNA interference and control of cell cycle functions [18].

ceRNA activity was also reported as a mediator of host-virus interaction in virus infected cells. Cazalla and his group also reported that viral U-rich noncoding RNAs called HSUR expressed in primate virus herpesvirus saimiri (HVS) infected T cells are able to bind to three host miRNAs. They also noted that this activity resulted in striking alteration of the cellular levels of one of these miRNAs, miRNA-27. This phenomenon leads to the regulation of expression of the host-cell genes targeted by this miRNA [19].  Table 1 lists the ceRNAs found to be linked with various diseases. Recent advances about circular RNAs suggest that they can be possibly involved with diseases by their association with disease related miRNAs [29]. A recently published database (http://gyanxet-beta.com/circdb) circ2Traits stores the information about circular RNAs potentially associated with human diseases by their putative interactions with disease associated miRNAs [30].

## 5. Conclusion

Modelling the post-transcriptional regulation of gene expression by miRNAs has been a widely studied field of research through the last decade. But recently the discovery of a new player, which is an essential and integral part of this circuitry, has generated new interest in this field. This new player is named competing endogenous RNA or ceRNA as it competes for available miRNA pool in cells to impart effects on the miRNA-targets. The competing RNA can be another protein coding RNA or a non-protein-coding pseudogene or a long noncoding RNA or circular RNA. The intricate networks of ceRNAs in cells are a fascinating new subject of study for researchers working towards understanding the language of RNA molecules in the post-transcriptional regulation of gene expression. In a ceRNA network, all the components coregulate each other in a manner such that a small perturbation in any of the components would result in an out of equilibrium condition in the system. The ceRNAs can be connected to each other by direct or indirect connections (see Figure 1(a)); that is, two ceRNAs may share binding sites for a common miRNA (direct connection) or they can be connected through a third ceRNA (indirect connection). For modelling the effects of ceRNAs in the post-transcriptional regulation of gene expressions in cells, the effects of both directly and indirectly connected ceRNAs need to be considered. Also the effects of ceRNAs need to be modelled in the real scenario in cells where many miRNAs interact with many targets, not only a single miRNA interacting with some targets. Another important consideration will be the binding preference of miRNAs among its many targets as seen in Figure 1(d). It can be speculated that, in a network, the ceRNA having more binding preference to the shared miRNA will have more profound ceRNA effect on the components with less binding preference. A robust model consisting of all these features, accompanied by detailed experimentations for validating the effects of perturbation of different components in the model, should effectively depict the scenario of miRNA-mediated post-transcriptional regulation in cells.

ceRNAs have been reported as key regulators in many biological processes including cell cycle, tumor suppression, and development and found to be involved in several diseases including cancer. The general disruption of miRNA pathway in cancer [31] has profound bearing on the underlying ceRNA network. The ceRNA effects will unravel many new and unexplored pathways in cancer. The intricate involvement of miRNAs in stages of development implies that ceRNAs play an important part in developmental process. ceRNA is going to be the key for future studies on post-transcriptional control.

## Figures and Tables

**Table 1:** A list of ceRNAs reported to be associated with diseases.

ceRNAs	Shared targeting miRNA(s)	Involved in disease
PTEN and ZEB2	miR-181, miR-200b, miR-25, and miR-92	Melanoma [11]

PTEN and CNOT6L (miR-17, 19a, 19b, 20a, 20b, and 106b) VAPA (miRs 17, 19a, 20a, 20b, 26b, 106a, and 106b)	miRs 17, 19a, 20a, 20b, 26b, 106a, 106b, and 19b	Prostate cancer [12]

PTEN and 13 ceRNAs including RB1, NRAS, KLF6, HIF1A, HIAT1, CTBP2, and TNKS2	It is predicted that PTEN and RB1 share MRE for 32 common miRNAs [7]	Glioblastoma [13]

CD44 and CDC42	miR-216a, miR-330, and miR-608	Breast cancer and many other cancers [14]

Versican and Rb1 PTEN	miR-199a-3p and miR-144	Breast cancer [15]

PTEN and PTENP1	miR-19b and miR-20a	Controls downstream PI3K signalling and cell proliferation in several cancers [8]

PRKACB and HULC	miR-372	Hepatocellular carcinoma [16]

PTCSC3 and targets of miR-574-5p	miR-574-5p	Thyroid cancer [17]

LMNA and 17 ceRNAs (predicted)	11 miRNAs including miR-9, miR-34a, miR-539, miR-449a, and miR-671 [16]	Hutchinson-Gilford progeria syndrome [18]

HSUR (HVS)	miRNA-27	Controls host-virus interaction in HVS infected T cells [19]

## Abbreviations

ceRNA: Competing endogenous RNA
mRNA: Messenger RNA
miRNA: microRNA
MRE: microRNA response element
lncRNA: Long noncoding RNA
circRNA: Circular RNA
ceRNET: ceRNA network.
